# Leagues of their own: sexually dimorphic features of meiotic prophase I

**DOI:** 10.1007/s00412-019-00692-x

**Published:** 2019-03-02

**Authors:** Cori K. Cahoon, Diana E. Libuda

**Affiliations:** Institute of Molecular Biology, Department of Biology, University of Oregon, 1370 Franklin Boulevard, Eugene, OR 97403-1229 USA

**Keywords:** Meiosis, Recombination, Sexual dimorphism, Synaptonemal complex, Chromosome architecture, Crossing over, Crossover, Sex-specific differences, Chromosome axis, Spermatogenesis, Oogenesis, Germ cell development, Gametogenesis

## Abstract

Meiosis is a conserved cell division process that is used by sexually reproducing organisms to generate haploid gametes. Males and females produce different end products of meiosis: eggs (females) and sperm (males). In addition, these unique end products demonstrate sex-specific differences that occur throughout meiosis to produce the final genetic material that is packaged into distinct gametes with unique extracellular morphologies and nuclear sizes. These sexually dimorphic features of meiosis include the meiotic chromosome architecture, in which both the lengths of the chromosomes and the requirement for specific meiotic axis proteins being different between the sexes. Moreover, these changes likely cause sex-specific changes in the recombination landscape with the sex that has the longer chromosomes usually obtaining more crossovers. Additionally, epigenetic regulation of meiosis may contribute to sexually dimorphic recombination landscapes. Here we explore the sexually dimorphic features of both the chromosome axis and crossing over for each stage of meiotic prophase I in *Mus musculus*, *Caenorhabditis elegans*, and *Arabidopsis thaliana*. Furthermore, we consider how sex-specific changes in the meiotic chromosome axes and the epigenetic landscape may function together to regulate crossing over in each sex, indicating that the mechanisms controlling crossing over may be different in oogenesis and spermatogenesis.

Sexually reproducing organisms pass on genetic information to the next generation through the production of haploid gametes, such as sperm and eggs. Sperm and egg development initiates with the formation of primordial germ cells during embryogenesis (reviewed in Spiller et al. 2017). Proliferation and expansion of these germ cells create a pool of germline stem cells. The germline stem cells divide asymmetrically to generate either more stem cells or differentiated germ cells. Differentiated germ cells transition from mitosis to meiosis and sexually differentiate to develop into either eggs or sperm (Table 1).

**Table 1:** Characteristics of eggs and sperm in humans

	Oocyte	Sperm	Reference
Size	Largest cell (~ 4,000,000 μm^3^)	Smallest cell (~ 30 μm^3^)	(Kleinhans et al. 1992; Goyanes et al. 1990)
Number	∼ 20 weeks gestation: several million At birth: ∼ 1–2 million At 37.5 years old: ∼ 25,000 At 51 years old: 1000 oocytes	Hundreds of millions	(Lobo 2003)
Temperature	Basal body temperature	2–4 °C below basal body temperature	(Kim et al. 2013)
Produced	During fetal development	Starting at puberty and continuously throughout the lifespan	(Morelli and Cohen 2005)
Arrest	Dictyate arrest at late prophase I until puberty	None	(Nagaoka et al. 2012; Morelli and Cohen 2005; Hunter 2017)
Aneuploidy rates	10–70%	1–4%	(Nagaoka et al. 2012; Hunter 2017)

Meiosis produces gametes, such as sperm and eggs, with exactly half the number of chromosomes as the original parent germ cell. To ensure a successful meiosis in most organisms, there are three events that must occur: (1) homologous chromosomes must pair; (2) homologous chromosomes must repair double-strand DNA breaks (DSBs) to form crossovers, which forge a physical connection between the chromosomes; and (3) homologous chromosomes must undergo two successive segregation events. A crossover allows for accurate segregation of the homologs during meiosis I. Thus, all the preceding steps to the formation of a crossover are highly regulated to guarantee that at least one crossover is formed between each pair of homologous chromosomes. As part of the crossover regulation process, crossovers undergo a “designation” to limit the number of DSB sites licensed to mature into crossovers (Yokoo et al. 2012). When errors occur during meiosis, the resulting gametes are frequently aneuploid, with either too many or too few chromosomes. In humans, meiotic errors are the leading causes of miscarriages and birth defects (reviewed in Nagaoka et al. 2012).

Both oogenesis and spermatogenesis produce a haploid gamete containing a complete complement of heritable genetic information. However, a growing body of evidence indicates that meiotic mechanisms differ between oogenesis and spermatogenesis. One of the largest mechanistic differences between oogenesis and spermatogenesis is the developmental timing and duration of when each process is occurring (Tables 1 and 2). In mammals, oogenesis occurs during fetal development in utero, with oocytes eventually arresting and maintained in late prophase I for decades. A majority of the defects that occur in human oocytes happen during this lengthy arrested stage with older oocytes having a higher chance of being aneuploid due to issues such as protein degradation (reviewed in Nagaoka et al. 2012).

**Table 2:** Summary of developmental and chromosomal contexts of meiosis between organisms

	Sexes	Reproductive organ	Timing of meiosis	DSB formation	Chromosome classification (centromere position)
*Mus musculus*	Male	Testes	Starts at puberty and occurs throughout lifespan	Required for homolog pairing and occurs prior to synaptonemal complex assembly	Acrocentric
	Female	Ovary	During fetal development		
*Caenorhabditis elegans*	Male	Gonad	Occurs throughout lifespan	Not required for homolog pairing and occurs within the context of assembled synaptonemal complex	Holocentric
	Hermaphrodite		Larval L4 stage undergoes both oogenesis and spermatogenesis then, at adulthood, switches to only oogenesis which continues throughout lifespan		
*Arabidopsis thaliana*	Male	Anthers	Annual plant (flowers after ~ 3 weeks)	Required for homolog pairing and occurs prior to synaptonemal complex assembly	Metacentric and acrocentric
	Female	Ovaries			

In contrast to oogenesis, spermatogenesis in mammals occurs throughout the lifespan of the organism (Tables 1 and 2). While the quantity of sperm produced does decrease with time, no single sperm is stored for decades as is found with oocytes (Morelli and Cohen 2005). Notably, in organisms where both spermatogenesis and oogenesis occur at similar developmental time periods (e.g., throughout the lifespan of the adult organism; Table 2), the process of spermatogenesis is executed faster than that of oogenesis. In *Caenorhabditis elegans*, while meiotic prophase I of spermatogenesis is completed in 20–24 h in the adult male, meiotic prophase I of oogenesis requires 54–60 h to complete in the adult hermaphrodite (Jaramillo-Lambert et al. 2007). Similarly, in Drosophila, spermatogenesis occurs in ~ 5 days, but oogenesis takes ~ 12 days, with six of these days being spent in early to mid-pachytene (Gyuricza et al. 2016; Lindsley and Tokuyasu 1980).

Another large difference between spermatogenesis and oogenesis is the strength of the checkpoint monitoring for errors in both DNA damage and chromosome segregation. It has been well established that during oogenesis the response of checkpoints to errors is very poor, whereas the checkpoints during spermatogenesis are so robust that errors are extremely rare (Morelli and Cohen 2005). Numerous studies have investigated the differences between the checkpoint responses in spermatogenesis and oogenesis and these findings have been extensively reviewed in Morelli and Cohen 2005.

Additionally, the end products of oogenesis and spermatogenesis are very different: egg and sperm, respectively. During oogenesis, the chromosomes undergo an asymmetrical division with half the genome being segregated into polar bodies producing only a single viable gamete, which is the largest cell type in humans. In contrast, spermatogenesis undergoes symmetrical divisions during meiosis making four viable gametes, which is the smallest cell type in humans. Additionally, recent evidence suggests that the large size of the oocyte may contribute to the high frequency of chromosome missegregation (Kyogoku and Kitajima 2017). Thus, multiple factors may be contributing to the error-prone nature of oocytes in humans.

While both oogenesis and spermatogenesis need to undergo all the steps of meiotic prophase I, the mechanisms of how homologous chromosomes pair, synapse, and recombine appear to be different for each process. In this review, we discuss the differences between oogenesis and spermatogenesis at each stage of meiotic prophase I: leptotene, zygotene, pachytene, diplotene, and diakinesis. Specifically, we focus on the sex-specific differences in the establishment and formation of the chromosome architecture and position and distribution of crossovers in three model organisms where these sexually dimorphic features are most well characterized: *Mus musculus*, *Caenorhabditis elegans*, and *Arabidopsis thaliana* (Tables 2 and 3).

**Table 3:** Summary of sexually dimorphic features of meiosis

	Sex	Leptotene			Zygotene		Pachytene			Diplotene/diakinesis
		Axis length	Mutants	DSB	Synaptonemal complex	Mutants	Crossing over	Biased hotspots	Crossover distribution	DNA compaction
*Mus musculus*	Male	Shorter chromosome length	*rad21L* mutants are sterile and display axis assembly defects	No difference in DSB numbers	Wider SC width	*sycp2* and *sycp3* mutants are sterile	Less crossovers	Some hotspots are stronger (male-biased usage)	DNA methylation suppresses crossing over at female-biased hotspots and promotes crossing over at male-biased hotspots	DNA histones replaced with protamines
			*smc1β* mutants have axis defects and arrest at pachytene	TEX15 is critical for the loading of RAD51 and DMC1	C terminus of SYCP1 is next to the lateral element					
	Female	Longer chromosome length	*rad21L* mutants are fertile with an age-dependent fertility defects	No difference in DSB numbers	Shorter SC width	*sycp2* and *sycp3* mutants are subfertile	More crossovers	Some hotspots are stronger (female-biased usage)	Suppressed in distal regions even though locally there are more DSBs in this region than males	Unknown
			SMC1β mutants have axis defects and arrest at metaphase II	TEX15 is not required for the loading of RAD51 and DMC1	C terminus of SYCP1 is inserted into the lateral element					
				5′ end of the DSB is resected more						
*Caenorhabditis elegans*	Male	Unknown	*rec-8* mutants are subfertile	Unknown	Unknown	Unknown	Unclear, but possibly less crossing over	Unknown	Double crossovers occur closer together	DNA histones replaced with protamine-like proteins
	Hermaphrodite	Unknown	*rec-8* mutants only have minor fertility defects	Unknown	Unknown	Unknown	Unknown	Unknown	More crossovers in the medial areas	Unknown
									Crossing over is suppressed at the chromosome ends	
									Positions of crossovers vary on each autosome	
									Double crossovers are as far apart as possible	
*Arabidopsis thaliana*	Male	Longer chromosome length	*syn3* mutants cause pairing and synapsis defects with only a slight reduction in pollen viability	Unknown	Unknown	Unknown	More crossovers	Yes (male only hotspots)	DNA methylation influences placement of crossovers	MGH3 is a male-specific H3 variant that is suspected to function similar to protamines in the development of male gametes.
			*scc2* mutants are sterile							
	Female	Shorter chromosome length	*syn3* mutant cause pairing, synapsis, and chromosome segregation defects resulting in sterility	Unknown	Unknown	Unknown	Less crossovers	Yes (female only hotspots)	Suppressed in distal regions	Unknown
			*scc2* mutants are subfertile							

## Leptotene: formation of the meiotic chromosome axes and double-strand DNA breaks

**Fig. 1:**
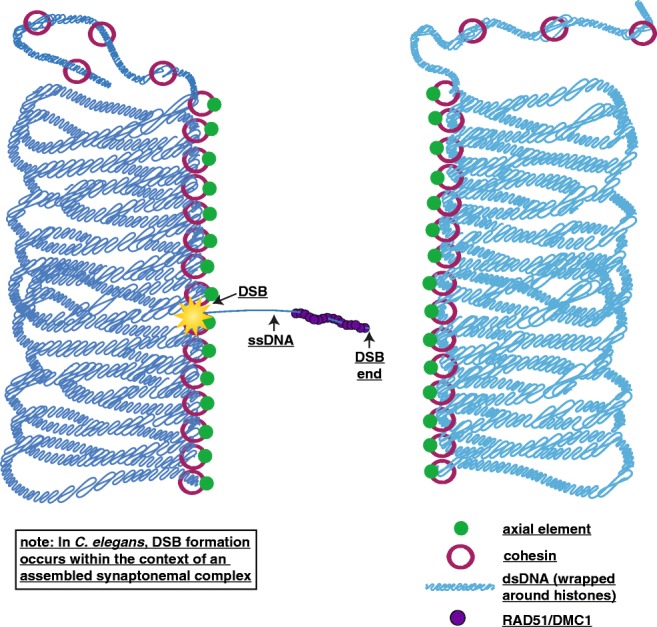
Leptotene. During leptotene, meiotic chromosomes begin to organize into a loop-axis DNA structure where loops of DNA extend out from a chromosome axis. Depicted is a pair of homologous chromosomes in dark and light blue with the lines of the chromosomes representing double-strand DNA (dsDNA) wrapped around histones. For simplicity, only one sister chromatid of each homolog is shown. The loop-axis structure is formed by cohesins (maroon rings) and axial elements (green). To facilitate homolog pairing and form crossovers, double-strand DNA breaks (DSBs, represented by the yellow star) are made and resected to reveal a region of single-strand DNA (ssDNA). This single-strand DNA is coated in the recombinase proteins RAD51/DMC1 (purple) and underdoes a search process for homologous sequences. For simplicity, the other end of the DSB is not depicted, but it will also be resected and coated in RAD51/DMC1. In *C. elegans*, homolog pairing occurs prior to the formation of DSBs and DSBs are made within the context of assembled synaptonemal complex

Leptotene, the first stage of meiotic prophase, begins following meiotic DNA replication with a reorganization of the chromatin into the meiotic loop-axis structure where loops of DNA extend out from a central chromosome axis (Fig. 1) (reviewed in Zickler and Kleckner 1999; Mercier et al. 2015). During mitosis, it has been shown that cohesins are critical to create loops for compaction and organization of the chromatin (Alipour and Marko 2012; Goloborodka et al. 2016). Thus, the meiotic loop-axis structure is established by a group of cohesin and cohesin-like proteins known as axial elements. Although it is unclear why meiotic chromatin assumes this loop-axis arrangement, one postulation is that this arrangement provides some stiffness and organization to the chromosomes to facilitate homolog pairing and recombination (Zhang et al. 2014). Nevertheless, this loop-axis structure is critical for the formation and placement of crossovers later during meiotic prophase I progression.

Following the establishment of the meiotic chromatin structure, homologous chromosomes must identify each other and pair. In some organisms, like yeast, *Arabidopsis*, and mice, the process of identifying the homolog is initiated by the formation of programmed DSBs (reviewed in Zickler and Kleckner 2015; Mercier et al. 2015) (Table 2). These DSBs facilitate homology search by creating a single-strand section of DNA that searches for the appropriate homologous sequence (or repair template) on other chromosomes. In other organisms, homologous chromosome pairing is initiated by specific proteins called pairing center proteins (*C. elegans*), or by chromatin states (Drosophila) (Dernburg et al. 1996; MacQueen et al. 2005; Phillips et al. 2009) (Table 2).

Since the mechanisms of homolog pairing still remain largely unclear, the sexually dimorphic properties and mechanisms of pairing are also not well understood. Mutant analyses from different organisms suggest that organisms have evolved multiple ways to pair homologous chromosomes (Zickler and Kleckner 2015, 2016; Mercier et al. 2015). Nevertheless, even for those organisms, such as *C. elegans*, where the proteins involved are known, it is not clear how these proteins and the chromosome-wide chromatin states coordinate this pairing process (MacQueen et al. 2005; Phillips et al. 2009; Wynne et al. 2012; Rog and Dernburg 2013; Dombecki et al. 2011).

Sex-specific differences have been identified in both the establishment of the meiotic loop-axis structure and the processing of DSBs. While both spermatogenesis and oogenesis assemble the meiotic loop-axis structure, the chromosomes do not end up being the same lengths upon the completion of this process. Further, both spermatogenesis and oogenesis similarly initiate the formation of DSBs with the conserved endonuclease Spo11, but how these programmed DSBs are both resected and processed in each sex is different (Baudat et al. 2000; Grelon et al. 2001; Dernburg et al. 1998). Currently, the most comprehensive studies identifying sex-specific differences in chromosome axes and DSB processing have been done in mice; thus, our discussion will begin with *Mus musculus*.

### *Mus musculus*

In mice, as well as humans, females have longer meiotic chromosome axes than males (Gruhn et al. 2013; Morelli and Cohen 2005). While it remains unknown why females have longer chromosome axes, mutant analyses of axis proteins indicate that there are sex-specific differences in the requirements for specific axial proteins during meiosis. Thus, a difference in the organization of the axis proteins might provide an explanation for these differences in chromosome axis lengths.

One of the axial proteins that displays sex-specific phenotypes upon knockout is the meiotic cohesin protein RAD21L. Specifically, RAD21L is required for chromosome axis formation and fertility in males, but not in females (Herran et al. 2011). Instead, *rad21L* mutant females display no defects in meiotic chromosome axis formation; however, they do display an age-dependent fertility defect that might be caused by a rapid depletion of oocytes (Herran et al. 2011).

An additional axial protein, SMC-1ß, also displays sex-specific differences upon knockdown, with males arresting in the middle of prophase I and females displaying sister chromatid cohesion defects at metaphase II (Revenkova et al. 2004). However, unlike *rad21L* mutants, *smc1-ß* mutants cause similar defects in chromosome organization in both males and females, with both exhibiting reduced axis lengths and recombination defects (Revenkova et al. 2004). Taken together, the delay in meiotic failure for females likely reflects the less restrictive checkpoint control of oogenesis (reviewed in Morelli and Cohen 2005).

Other known meiotic cohesin and cohesin-like proteins, such as REC8 and STAG3, both display very similar phenotypes between males and females. When either *rec8* or *stag3* is mutated, both sexes are infertile due to defects in homolog pairing and recombination (Winters et al. 2014; Caburet et al. 2014; Fukuda et al. 2014; Xu et al. 2005). Additionally, double mutant analysis of *rec8* or *rad21L* with *stag3* in males suggests that these proteins might function as distinct complexes at different regions of the chromosomes (Ward et al. 2016). While REC8/STAG3 seem act as the primary centromeric cohesin complex (Ward et al. 2016), the RAD21L cohesin complexes promote heterochromatic clusters or “chromocenters” and may facilitate DSB-independent homolog pairing (Ward et al. 2016; Ishiguro et al. 2014). Thus, the organization of the chromosomes by these cohesins may play a larger role in homolog pairing than was previously thought. Notably, the REC8/STAG3 cohesin complexes have not been extensively studied in female mice, where RAD21L is not required to establish the meiotic axis structure (Herran et al. 2011). These sex-specific differences in the requirement for RAD21L bring up intriguing possibilities that (1) chromosome axis–driven homolog recognition may be different between males and females, or (2) this form of homolog pairing might be a male-specific feature of meiosis.

Following the establishment of the meiotic chromosome axes, programmed DSBs are formed similarly in males and females, but the initial processing of these DSBs differs. DSBs are created by the topoisomerase-like protein SPO11, and the 5′ end of the DNA is resected to reveal a short single-strand section of DNA that is then coated in the single-strand DNA binding recombinases RAD51 and DMC1 (Baudat et al. 2000; Tarsounas et al. 1999; Hunter 2015; Zickler and Kleckner 2015). Both of these proteins facilitate the repair of these DSBs as either crossovers or noncrossovers in both males and females (reviewed in Hunter 2015). Interestingly, the early DSB processing protein TEX15 is critical for loading of DMC1 and RAD51 in only males (Yang et al. 2008). *tex15* mutant females display no defects in loading DMC1 or RAD51 (Yang et al. 2008). Additionally, mice heterozygous for a dominant *dmc1* mutant that is unable to facilitate recombination abolishes recombination only in males, with young, heterozygous mutant females having no defects in crossing over (Bannister et al. 2007). Furthermore, single-strand DNA sequencing methods suggest that males resect back the 5′ end of the DSB more than females (Brick et al. 2018). Taken together, the differences between males and females in both the processing of DSBs and the requirement of specific recombination proteins may indicate different mechanisms of repair between the sexes.

Sex-specific differences in mutant phenotypes are a reoccurring theme in mouse meiosis; therefore, it is important to analyze mutant phenotypes in both males and females (Morelli and Cohen 2005). The sexually dimorphic features of the chromosomes axis have revealed that the organization of the axial elements may be different between the sexes. Furthermore, these distinct axial organizations might generate different recombination landscapes in spermatocytes and oocytes.

### *Caenorhabditis elegans*

Similar to mice, worms also display sex-specific differences in chromosome axis proteins. Mutant males for the kleisin cohesin protein REC-8 have highly aneuploid sperm that cause severe defects in fertility (Severson et al. 2009). In comparison, *rec-8* mutant oocytes display only slight defects in fertility, likely due to the presence of other cohesin proteins COH-3 and COH-4 which compensate for the loss of REC-8 (Severson et al. 2009; Severson and Meyer 2014). Currently, it is unknown whether *coh-3* and *coh-4* mutants cause male infertility, but oocytes that are triple mutant for *rec-8*, *coh-4*, and *coh-3* are infertile (Severson et al. 2009, Severson and Meyer 2014). Together, all three cohesin proteins are required for sister chromatid cohesion and axis formation during oogenesis.

Although worms display sex-specific differences in the requirement for certain chromosome axis proteins, it is unknown if oocytes and spermatocytes in worms have different chromosome axis lengths. It is intriguing to speculate that worms could be establishing different axes in each sex, which might cause sex-specific changes in the chromosome axis length. Moreover, these sex-specific dependencies on certain axis proteins might set up a foundation for sex-specific changes in DSB processing and repair. Currently, it is unclear in worms if spermatocytes and oocytes display differences in DSB formation and processing. However, studies in oocytes have started to shed light on the mechanisms behind DSB formation and processing, and these mechanisms are discussed in a recent review (Yu et al. 2016).

### *Arabidopsis thaliana*

In *Arabidopsis thaliana*, males have longer chromosome axes than females (Drouaud et al. 2007). Similar to mice, it is unclear why one sex forms longer chromosome axes. Notably, this increase in chromosome length does correlate with an increase in crossing over (see the “Pachytene: establishment of crossovers between homologs” section below). Although the sex with the longer chromosome axes varies among species, sex-specific differences in chromosome length may be a conserved feature of meiosis.

Similar to worms and mice, *Arabidopsis* displays sex-specific differences in the cohesin proteins that assemble the chromosome axis. The meiosis-specific kleisin REC8, also known as SYN1, has three other closely related proteins (SYN2, SYN3, and SYN4). SYN2 and SYN4 are thought to function in mitotic cells. Knockdown of *syn3* results in sex-specific differences, with female *syn3* mutants displaying more severe defects than male *syn3* mutants (Yuan et al. 2012). In both sexes, knockdown of *syn3* causes delays in chromosome pairing and partial synapsis, but only female *syn3* mutants have aberrant chromosome segregation (Yuan et al. 2012). Knockdown of SYN3 results in complete termination of the oogenesis process, whereas males produce pollen with only a slight reduction in pollen viability (Yuan et al. 2012). However, RNAi knockdown is highly variable in *Arabidopsis* and studies have shown that genetic mutants may not always phenocopy the protein knockdown mutants (Siaud et al. 2004; Li et al. 2004). Nonetheless, these studies reaffirm the importance in assaying mutants in both sexes.

Sex-specific differences in fertility are also found upon knockdown of the cohesin subunit, SCC2. Male *scc2* mutants are completely sterile, while female *scc2* mutants are only partially sterile (Sebastian et al. 2009). This fertility difference could be explained by incomplete knockdown of SCC2 in females, but it is also possible that females depend differently on SCC2 than males during meiosis. However, due to the caveats mentioned above about RNAi knockdown in *Arabidopsis*, future experiments are necessary to determine if females do have a less severe defect in the SCC2 mutants.

Differences in the requirement for axis proteins may cause changes in chromosome axis organization, thereby resulting in the different lengths of the chromosome axes between males and females. Furthermore, changes in the organization of the axis might also influence recombination since the sex that has the longer axis has more crossing over. Thus, future experiments examining the organization of the axis proteins and how this organization relates to downstream repair events might reveal how the chromosome axis directs DSBs and recombination.

### Zygotene: initiation of synaptonemal complex assembly

**Fig. 2:**
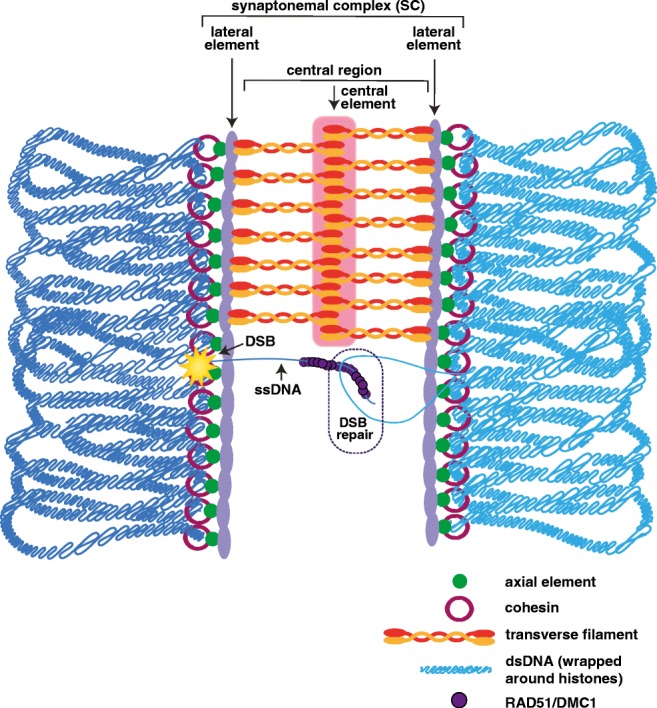
Zygotene. At zygotene, homologous chromosomes (dark and light blue lines that represent double-strand DNA wrapped around histones) have paired and the synaptonemal complex (SC) starts to assemble. The SC consists of three parts: lateral elements, central region, and central element. The lateral elements (light purple) assemble alongside the axial elements (green) and cohesins (maroon rings). The central region contains the transverse filament proteins (red and orange), which span the distance between the two homologs and interact with the lateral elements. The central element (pink) contains a group of proteins that are thought to help to stabilize the middle of the SC. Although many of the steps of DSB repair are thought to occur during pachytene, some of the repair processes are initiated in zygotene. The dashed-circle region indicates a region where the single-strand DNA (ssDNA) coated in RAD51/DMC1 recombinase proteins (purple) is initiating DSB repair. The DSB is represented by the yellow star

Once homologous pairing has been established, the homologs begin to align and assemble the synaptonemal complex (SC), which initiates the transition from leptotene to zygotene (Fig. 2). The SC is a highly conserved, tripartite protein complex that functions to maintain homolog pairing and facilitate recombination (reviewed in Cahoon and Hawley 2016). The SC contains three regions known as the lateral elements, the central region, and the central elements (Fig. 2). The lateral element, also known as axial elements prior to the assembly of the SC, run along either homolog and connects the other SC proteins to the chromatin. The central region proteins lie within the middle region between the two homologs and contain the transverse filament proteins that create the bridge that links the two lateral elements together. The central elements are located in the center of the SC and are thought to provide support for the central region proteins. Additionally, recent evidence in both flies and worms demonstrated that the SC and the axis components are dual layered with each layer of SC connecting one sister chromatid of each homolog (Cahoon et al. 2017; Kohler et al. 2017).

In some organisms, such as mice and yeast, the assembly of the SC initiates at multiple regions along the chromosomes that corresponds with sites of DSBs (reviewed in Cahoon and Hawley 2016). In other organisms, like worms and flies, the SC assembles prior to the formation of DSBs (reviewed in Cahoon and Hawley 2016). Notably, SC assembly in flies, and possibly worms, still occurs from multiple regions along the chromosomes; however, it is unclear what is driving the assembly of the SC from the interstitial chromosomal sites (Tanneti et al. 2011; Nabeshima et al. 2011). Once SC assembly begins, the complex is rapidly built until the chromosomes are synapsed from telomere to telomere.

Similar to the axial elements, the SC also exhibits sexually dimorphic features. These sexually dimorphic features of the SC are likely due to the fact that the SC assembles upon axial elements with sexually dimorphic features. In particular, the lateral elements of the SC interact with the cohesins in the chromosome axis and many of the sex-specific differences in the SC involve the lateral element proteins. Although it is not known whether the SC of *C. elegans* or *A. thaliana* displays sexually dimorphic features, several lines of evidence in mice indicate sex-specific differences in the lateral element.

### *M. musculus*

In mice, two lateral element proteins, SYCP2 and SYCP3, display sex-specific phenotypic differences where mutants of either protein cause sterility in males and subfertility in females (Yuan et al. 2000, 2002; Yang et al. 2006, 2008). Additionally, FKBP6 is an SC protein that only causes defects in males, even though FKBP6 localizes to the SC in females (Crackower et al. 2003). Currently, the role of FKBP6 in female meiosis is unclear. Future experiments examining mutant phenotypes in both sexes are critically important because these sex-specific disparities in mutant phenotypes something that repeatedly occurs in mouse meiosis (Morelli and Cohen 2005).

*sycp2* and *sycp3* mutants exhibit altered assembly of the SC. In *sycp2* and *sycp3* mutant males, the SC fails to assemble (Yang et al. 2006; Yuan et al. 2002). However, in *sycp2* and *sycp3* mutant females, the SC partially assembles, crossover numbers are decreased, and fertility is partially reduced (Yang et al. 2006, Yuan et al. 2002). Interestingly, *sycp3* mutant females display an age-dependent fertility defect similar to that of *rad21L* mutants (Yang et al. 2006; Yuan et al. 2002). Notably, it is still unclear whether the *sycp3* mutant females also display a similar oocyte depletion phenotype as found in *rad21L* mutant females.

The ability for females to be subfertile in the absence of specific axial or lateral element proteins may be the result of the poor checkpoint response in oogenesis allowing for chromosome segregation when homolog pairing and/or recombination are defective. However, some of the axial and lateral element mutants display no defects in fertility suggesting that the structure and possibly the composition of the SC may be different in each sex. Since no sex-specific SC proteins have been identified yet, the composition of the SC appears to be the same between male and female mice (Agostinho et al. 2018). Despite this similar protein composition, the width or distance between the lateral elements of the SC is ~ 60 nm shorter in females than in males (Agostinho et al. 2018). This decrease in SC width in female mice is likely the result of positioning the transverse filament protein, SYCP1, deeper into the lateral element, thereby suggesting that the organization of the proteins within the lateral element may differ in females (Agostinho et al. 2018). Moreover, sex-specific differences in SC width is not unique to mice. In the silk worm, *Bombyx mori*, females have an SC width that is ~ 30–40 nm shorter than that in males (von Wettstein et al. 1984).

Currently, there are not any published studies in metazoans for how changes in the SC width may affect meiosis. However, in yeast, mutants that alter the width of the SC cause defects during meiosis leading to reduced spore viability (Sym and Roeder 1995). While there are some caveats with these experiments, these studies raise interesting questions about how the width of the SC might affect DSB repair. Since DSB repair occurs on the chromosome axis, a DSB must reach across the width of the SC to reach the homolog to enable repair as a crossover (Borde and de Massy 2013). In yeast, it has been shown that the Dmc1/Rad51-coated single-strand DNA can span distances up to 400 nm, which is much larger than the ~ 100-nm width of the SC (Brown et al. 2015). Thus, modulating the width of the SC could affect the efficiency or stability of a single-strand DNA molecule accessing the homolog as a repair template. Overall, ongoing studies may reveal connections between SC width and regulation of recombination.

## Pachytene: establishment of crossovers between homologs

**Fig. 3:**
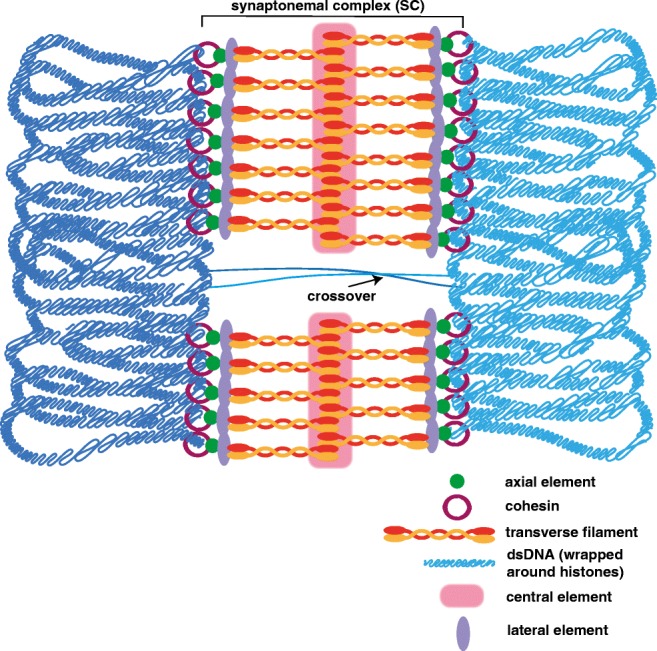
Pachytene. At pachytene, the homologs (dark and light blue lines that represent double-strand DNA wrapped around histones) have fully synapsed with the SC assembled from telomere to telomere. Both cohesin (maroon rings) and axial elements (green) are lost at the site of the DSB to facilitate in the repair of the break as either a crossover (depicted) or noncrossover (not depicted)

Fully synapsed chromosomes are a hallmark of the pachytene stage of prophase I, with synapsis being maintained until the end of this stage (Fig. 3). Additionally, during pachytene, most (if not all) DSBs are repaired as either crossovers of noncrossovers. Interestingly, an excess of DSBs is created to ensure that at least one of these breaks is repaired as a crossover per homolog pair. Thus, the fate of the DSB being processed as a crossover or noncrossover is a highly regulated process.

Many proteins are involved in whether a DSB is repaired as a crossover or a noncrossover; however, the exact mechanism of how specific DSBs are selected to become crossovers or noncrossovers is currently unclear (reviewed in Hunter 2015; Mercier et al. 2015). In many organisms, studies have shown that specific proteins are recruited to crossover-designated DSBs, but how those specific proteins are recruited to specific DSBs is unknown. Additionally, in mammals, the DNA binding site for the histone methyltransferase PRDM9 occurs near regions that will form crossovers, and these genomic regions are referred to as hotspots (Grey et al. 2018). Although PRDM9 adds open chromatin marks to histone H3 (mono-, di-, and trimethylation of K9 and K36), how PRDM9 directs crossovers to be formed at these locations is unclear (Eram et al. 2014; Koh-Stenta et al. 2014; Powers et al. 2016). A recent review on this topic examines the most current models for how PRDM9-directed crossover designation might occur (Grey et al. 2018).

Moreover, PRDM9 is not the only mechanism involved in crossover designation in mammals because a small fraction of crossovers will occur in regions with no PRDM9-binding sites, and it is unknown how DSBs in these regions are selected to be repaired as a crossover (reviewed in Gray and Cohen 2016). Since PRDM9 is not found outside of mammals, these non-PRDM9-marked crossovers might use an ancestral mechanism similar to how other organisms designate crossovers.

The SC, likely through recruitment of the DNA repair machinery, is highly involved in recombination both in the regulation of crossover distribution and in the repair of the DSB (Cahoon and Hawley 2016; Hunter 2015). Misregulation of SC assembly or disassembly results in alterations in crossing over (Cahoon and Hawley 2016; Libuda et al. 2013). Thus, the sex-specific differences found in the SC and the chromosome axes may factor into the regulation of recombination. In fact, sex-specific differences in crossover distributions have been found in many organisms (Bherer et al. 2017; Sardell et al. 2018; Johnston et al. 2017; Wellenreuther et al. 2013; Labonne et al. 2007; Burt et al. 1991; Singer et al. 2002; Bennett et al. 1986). Moreover, in humans, protein variants in both crossover proteins and SC proteins cause sex-specific changes in recombination (Halldorsson et al. 2019). For example, the same variant of the crossover protein RNF212 in humans is associated with the highest recombination rates in males and the lowest recombination rates in females, while a variant of the SC protein SYCP3 is associated changes in distal crossing over in only males (Kong et al. 2008; Halldorsson et al. 2019). Since the meiotic chromatin architecture is formed prior to recombination, it is intriguing to speculate that the differences in chromatin structure between males and females may be involved in the sex-specific differences in crossover distribution along the chromosome arms.

### *M. musculus*

In both mice and humans, where females have longer chromosome axes, females have more crossovers than males (Morelli and Cohen 2005; Gruhn et al. 2013). Notably, in mice, this increase in crossovers is not due to an overall increase in the number of DSBs (Brick et al. 2018; de Boer et al. 2015). Additionally, most of the recombination hotspots are utilized in both sexes, but there does appear to be sex-biased strength of the hotspots, with some hotspots being more active in one sex than the other (de Boer et al. 2015; Brick et al. 2018).

The regulation of these sex-biased hotspots may be controlled, at least partially, by the methylation state of the DNA. DNA is globally demethylated in the female germline, but not the male germline (Seisenberger et al. 2012). Additionally, in males, the PRDM9-binding sites are frequently methylated at male-biased hotspots with the region adjacent to the PRDM9 sites being methylated at the female-biased hotspots (Brick et al. 2018). Moreover, decreasing the amount of DNA methylation in males causes an increase in recombination at normally female-biased hotspots and reduction in male-biased hotspots (Brick et al. 2018). Thus, in males, DNA methylation suppresses crossing over at female-biased hotspots and promotes crossing over at male-biased hotspots. Similarly, in *Arabidopsis*, DNA methylation is also involved in crossover regulation (see below); therefore, DNA methylation–mediated targeting of crossover positions may be a conserved feature of recombination control. Future studies are needed to determine how DNA methylation is acting to regulate crossover position and how DNA methylation may be influencing meiotic chromatin architecture.

In female mice, DNA methylation does not regulate crossover distribution; thus, chromosome architecture may play a larger role in positioning crossovers. An interesting feature of sex-biased hotspots is that they are arranged in clusters along the chromosomes and the usage of these clustered hotspots is not dependent on the DSB initiation machinery (Brick et al. 2018). Instead, it is likely that the sex-specific differences in meiotic chromosome architecture are playing a role in clustering these sex-biased hotspots along the chromosomes. Thus, female mice may rely on the underlying chromosome axis architecture to regulate the position of crossovers. Also, spatial clustering of sex-biased hotspots occurs in humans as well, which raises the possibility that the mechanism regulating this sex-specific clustering might be conserved (Gruhn et al. 2013; Bherer et al. 2017).

In both mice and humans, females suppress crossing over in the distal regions of the chromosomes despite the fact that females tend to have more DSBs in this region than males (Bherer et al. 2017; de Boer et al. 2015; Brick et al. 2018). Distal crossovers are known to be problematic for meiotic chromosome segregation, since homologs with these distal crossovers have difficulty aligning properly along the meiotic spindle (Ross et al. 1996). Considering that oocytes undergo a long late prophase I arrest, distal crossovers might predispose oocytes to segregation defects, thereby resulting in aneuploid eggs. To circumvent this chromosome segregation issue, oocytes suppress distal crossovers. In contrast, males do not undergo a long late prophase I arrest; therefore, some distal crossovers may be more tolerable. Furthermore, clustering of these sex-biased hotspots in females may provide a mechanism to regulate where crossovers are positioned. More studies are needed to determine if the chromosome axis structure influences the position of crossovers in females.

### *C. elegans*

In worms, it is unclear if sex-specific differences in recombination alter the number of crossovers (Hodgkin et al. 1979; Henzel et al. 2011; Gabdank and Fire 2018). The studies looking at sex-specific differences in crossing over are largely conflicting, with some studies suggesting a decrease in map length in males, while others indicate no change in map length between the sexes (Zetka and Rose 1990; Meneely et al. 2002; Lim et al. 2008). Further, the differences between these studies vary not only in the genetic backgrounds being assayed, but also in the method used to detect crossovers, with some studies using genetic markers and others using changes in single nucleotide polymorphisms between two haplotypes (Zetka and Rose 1990; Lim et al. 2008; Meneely et al. 2002). Moreover, each study differed by the particular chromosomes assayed for crossing over and by the total progeny scored, which varied from approximately 100 worms to thousands of worms (Zetka and Rose 1990, Lim et al. 2008, Meneely et al. 2002). Taken together, these differences between these studies may have led to the conflicting conclusions about the possibility of sex-specific differences in crossing over, or (as was found in the distal regions of mouse chromosomes) may reflect differences in crossing over between specific chromosomes or chromosomal loci.

Whole-genome sequencing using two isogenic strains has provided a highly detailed map of the position of the crossovers on all six chromosomes in hermaphrodites (Rockman and Kruglyak 2009). This study showed that recombination is largely suppressed in the central and distal chromosomal regions and enriched in what is considered the chromosome “arms” in *C. elegans* (Rockman and Kruglyak 2009), although the position of these crossovers varies widely on the autosomes (Rockman and Kruglyak 2009). Moreover, multiple studies have shown that during oogenesis, DSB formation and crossing over requires histone acetylation and methylation modifications (Wagner et al. 2010; McClendon et al. 2016; Bessler et al. 2007, 2010; Reddy and Villeneuve 2004). Currently, it is unclear whether post-translation modifications of histones are required for DSB formation and crossing over during spermatogenesis. Overall, many factors appear to be involved in regulating crossing over and it is important to assay whether recombination mutants cause defects to similar degrees in both sexes.

Currently, we do know whether the distribution of crossovers in worms is different between oogenesis and spermatogenesis. When double crossovers occur, spermatocytes tend to position these crossovers closer together than in oocytes (Lim et al. 2008). This comparative result suggests that crossover interference, which prevents the formation of crossovers near each other, is not as strong in spermatocytes as it is in oocytes (Muller 1916; Hodgkin et al. 1979; Henzel et al. 2011; Gabdank and Fire 2018). Additionally, if spermatocytes and oocytes do have the same number of crossovers, then this suggests that crossover distribution may be regulated differently than crossover designation.

### *A. thaliana*

In *Arabidopsis*, males have longer chromosome axes and more crossovers than females (Drouaud et al. 2007; Vizir and Korol 1900). Additionally, the distribution of crossovers differs in both males and females. Similar to female mice, *Arabidopsis* females suppress crossing over in the distal regions of the chromosomes, while *Arabidopsis* males display abundant crossing over in the telomeric regions (Giraut et al. 2011; Brick et al. 2018; de Boer et al. 2015). Thus, females must have an underlying mechanism that is influencing the DSB repair choice to strongly bias toward a noncrossover in these distal chromosomal regions.

Unlike mice, males and females in *Arabidopsis* display a sex-biased placement of crossovers. Surprisingly, only two elevated recombination regions, defined as hot regions, are used by both sexes (Giraut et al. 2011). In males, most of these hot regions are located near the telomeric regions, but DSBs are not strongly enriched in these regions (Choi et al. 2018; Giraut et al. 2011). Thus, it is possible that in males, the number of DSBs and the presence of a crossover may not be correlated. In females, most of the hot regions are in the pericentromeric regions of the chromosomes and the relationship between crossing over and number of DSBs remains unknown in females (Giraut et al. 2011). Currently, it is unclear why males and females utilize different regions; however, differences in the chromosome axes may be affecting the usage of each region in each sex.

Unlike male mice and male humans, neither male nor female crossover distributions in *Arabidopsis* correlate with the GC content of the DNA (de Boer et al. 2015; Clement and Arndt 2013; Arbeithuber et al. 2015). Instead, hot regions in *Arabidopsis* males tend to correlate with AT-rich regions, which are positioned at transcription start and termination sites (Choi et al. 2013; Drouaud et al. 2013). Overall, at a DNA sequence level, the positioning of recombination differs between organisms.

Although the epigenetic regulation of recombination may be similar between males in *Arabidopsis* and mice, DNA methylation in *Arabidopsis* inhibits crossing over in the repeat-rich heterochromatic regions and at specific euchromatic hotspots (Yelina et al. 2012; Yelina et al. 2015; Choi et al. 2013). Likewise in male mice, DNA methylation also suppresses the formation of crossovers at certain hotspots, specifically the female-biased hotspots (Brick et al. 2018). In contrast to mice, DNA methylation in *Arabidopsis* does not appear to promote hotspot activity. Thus, some other mechanism is acting in *Arabidopsis* to promote crossing over at the unmethylated hotspots.

One possible mechanism for crossover distribution in *Arabidopsis* is that DNA methylation may be influencing the organization of the chromosome axis. Surprisingly, a reduction in DNA methylation does not result in a large increase in crossing over in the euchromatin, but instead causes decreases in pericentromeric and centromeric crossovers (Yelina et al. 2012, 2015). Further, it has been recently found that reducing DNA methylation results in the inappropriate formation of DSBs in the centromeric heterochromatin and may explain the increase in centromeric recombination (Choi et al. 2018; Underwood et al. 2018). Thus, DNA methylation alone is not responsible for regulating euchromatic crossovers, but it strongly influences crossing over near the centromeres possibly by limiting the formation of DSBs in the centromeric heterochromatin. However, regardless of the amount of DSBs, a loss in DNA methylation downregulates the formation of crossovers (Choi et al. 2018, Underwood et al. 2018). Thus, DNA methylation may be involved in the designation of which DSBs become competent to form a crossover. All of the studies looking at the effects of DNA methylation on recombination have only been performed in males; therefore, it is unclear in females whether a similar type of regulation occurs. Future studies investigating DNA methylation in females may reveal sexual dimorphism of DNA methylation in addition to providing insight toward female-specific suppression of crossing over in the telomeric regions.

Sex-specific differences in recombination have also been recently identified in humans. Whole-genome sequencing studies in humans have found that females display more crossing over than males and, similar to other eukaryotes, recombination in females is suppressed in distal chromosomal regions (Halldorsson et al. 2019; Kong et al. 2008; Bherer et al. 2017). Surprisingly, the number of crossovers increase as maternal oocytes age, thereby suggesting a link between the maternal age effect and crossover number (Halldorsson et al. 2019). Further, epigenetic modifications also influence the placement of crossovers in humans (Halldorsson et al. 2019). Since many of the human datasets are from only one sex, it remains unclear if these epigenetic modifications display sex-specific patterns that alter recombination. Taken together, while sex-specific recombination patterns are conserved feature of meiosis, the function and mechanism behind patterning this sex-specific recombination landscapes are unknown.

## Diplotene and diakinesis: disassembly of the synaptonemal complex and condensation of the chromosomes

**Fig. 4:**
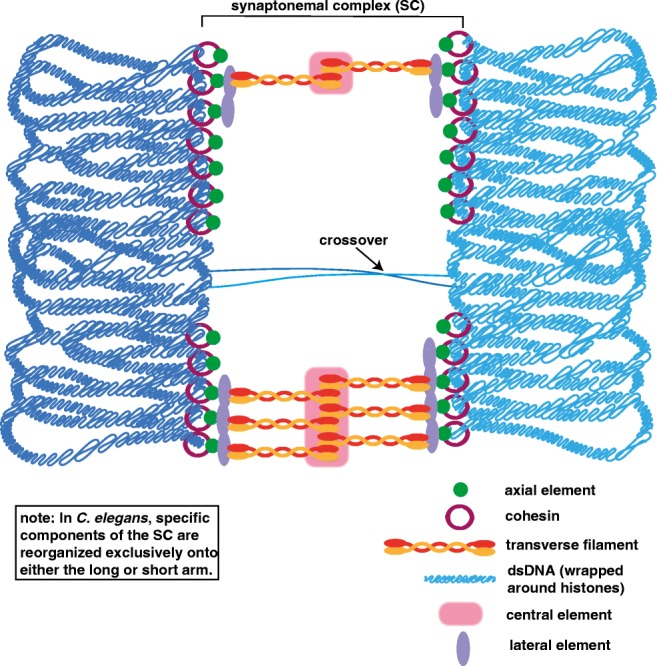
Diplotene. Diplotene begins after the repair of the DSBs with the disassembly of the SC. The cohesins (maroon rings) and axial elements (green) are not disassembled and likely help in organizing the chromosomes in diakinesis. The homologous chromosomes are shown in light and dark blue with the lines representing double-strand DNA (dsDNA) wrapped around histones

Initiation of SC disassembly prompts the start of diplotene (Fig. 4). The disassembly of the SC is regulated in many organisms by post-translational modifications of the central region proteins, which prompts a reorganization of the lateral element proteins (reviewed in Cahoon and Hawley 2016). Furthermore, it is extremely important that this disassembly process is linked with recombination, such that the disassembly of the SC is only triggered upon the completion of recombination. Although the mechanism of crosstalk between completion of recombination and SC disassembly is unknown, it is known that the major cell cycle kinases, such as POLO, MAP, and Aurora B kinases, are likely involved in linking these two processes (Cahoon and Hawley 2016). Additionally, it is currently unknown if there are any sex-specific differences in the regulation of SC disassembly.

**Fig. 5:**
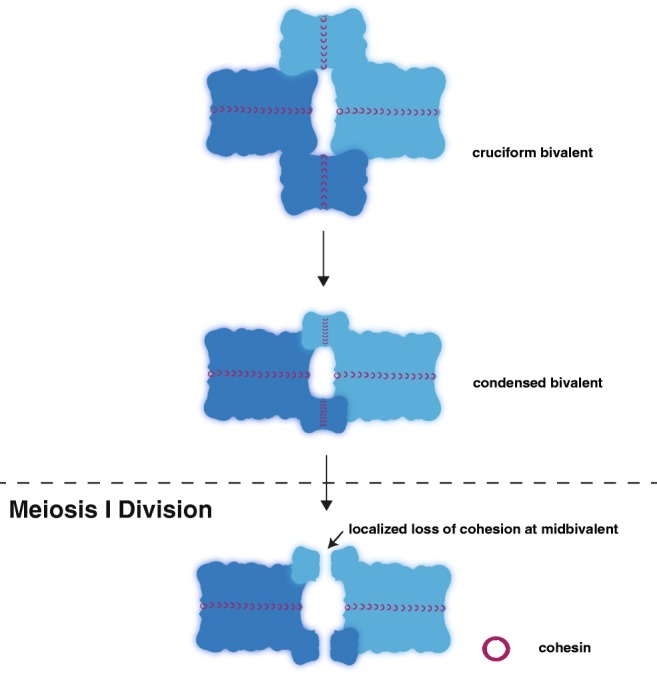
Diakinesis. The homologous chromosomes (dark and light blue) are rearranged during diakinesis to form a cruciform bivalent structure. This bivalent structure is then condensed by a group of proteins likely including cohesins, condensins, and DNA topoisomerases. At the meiosis I division, cohesin (maroon rings) is locally lost distal to the crossover site at the midbivalent allowing the homologs to separate. The residual cohesin is maintained between the sister chromatids until the second meiotic division when it is removed to allow the sisters to segregate (not depicted)

Following diplotene is diakinesis, the stage in which the chromosomes begin to condense (Fig. 5). In females, these condensed chromosomes form into cruciform or bivalent structures. However, in males, these bivalent structures are usually not visible due to both a higher degree of DNA compaction and a smaller nuclear volume in comparison to oocytes, although this compaction in *Arabidopsis* is reversed in the sexes, with female oocytes displaying a higher degree of DNA compaction and smaller nuclear volume than the pollen in males. This DNA compaction is accomplished in many organisms by the replacement of the DNA histones with histone alternatives. In mammals, DNA histones are gradually replaced with protamines throughout sperm maturation (reviewed in Sun and Handel 2008, Rathke et al. 2014), whereas in worms, the protamine-like proteins are exchanged in late meiotic prophase to compact the DNA (Chu et al. 2006; Shakes et al. 2009). Additionally, in *Arabidopsis*, a male-specific histone H3 variant, MGH3, is suspected to provide similar function to mammalian protamines (Okada et al. 2005). Notably, the mechanism of compacting of the homologs is unclear and likely involves cohesins, condensins, and DNA topoisomerases functioning together to compact the meiotic DNA (Hillers et al. 2017; Uhlmann 2016). Moreover, this compaction process may be similar to the compaction that occurs in mitotic cells, but the current models for mitotic chromatin compaction are controversial and sparse of mechanistic details (reviewed in Antonin and Neumann 2016). Taken together, aside from the higher degree of chromosome compaction caused by protamine replacement of histones in males, it is unknown if any of diakinesis-based chromosome compaction mechanisms are different between males and females.

## Summary and conclusions

Despite the fact in that both oogenesis and spermatogenesis produce haploid gametes, the mechanisms of how each meiotic stage enables the inheritance of genetic material are not the same. Moreover, these dissimilarities in meiosis span between organisms (Table 3). Mice and humans assemble longer chromosome axes in females, but in *Arabidopsis* it is the males that assemble the longer chromosome axes than females. These axis differences correlate with alterations in the recombination landscapes between males and females. Further, it is becoming apparent that the differences in the meiotic chromosome axis between the sexes may be driving the changes seen in recombination. In particular, the potential for crosstalk between epigenetic marks and the meiotic chromosome axis structure is an intriguing concept. Future studies looking at this intersection of DNA chemical and structural modifications might provide insights in how the chromosome axis may be direct or influence crossing over.

The reoccurring theme of meiotic axis mutants displaying sex-specific differences in mice, worms, and *Arabidopsis* supports a hypothesis that the axes between males and females are different. Future studies directly looking at the arrangement and composition of the axis proteins might provide mechanistic insights into the genetic differences observed with the meiotic axis mutants. Furthermore, it is unknown how these axis proteins are assembled and how dynamic these proteins are during meiosis. Understanding the mechanisms behind these genetic differences between the sexes may provide insights into how the chromosome axis is created and maintained throughout meiosis.

Stresses to the meiotic system, such as temperature increases, also reveal differences between oogenesis and spermatogenesis. For decades, meiosis has been known to be a temperature-sensitive process, in which temperature changes drastically affect both the position and number of crossovers as well as meiotic chromosome structures (reviewed in Morgan et al. 2017). Elevated temperatures are known to cause fertility defects in mammalian spermatogenesis (Paul et al. 2008; Durairajanayagam et al. 2015). Specifically, human males are particularly sensitive to narrow changes in temperature, with spermatogenesis requiring an isotherm 2–4 °C below basal body temperature (Kim et al. 2013). Although these temperature-induced effects on spermatogenesis are well known, the mechanisms behind these spermatogenesis-specific changes and sensitivities are not well understood.

In contrast to other model systems, temperature-induced changes during plant meiosis have been well studied. Since seasonal changes expose plants to a variety of environmental temperatures, it is extremely important to the agriculture industry to understand how temperature may be altering the seed or fruit production of a specific crop. In barley, increased temperature causes an increase in chromosome axis length and crossing over only in males (Phillips et al. 2015). Thus, males in barley, similar to mammalian males, are more sensitive to temperature changes. In *Arabidopsis*, males exposed to higher temperature display both a decrease in chromosome axis length, and (similar to barley) an increase in crossing over (Modliszewski et al. 2018; Lloyd et al. 2018). Future studies in *Arabidopsis* females are needed to determine if these responses to temperature are male-specific.

Increases in temperature also elevate recombination rates in Drosophila females (Grell 1973). Additionally, this increase in recombination correlates with decreases in fitness such that the number of progeny decreases as temperature increases (Grell 1973; Jackson et al. 2015). Also, studies in wheat observed that the grain number decreases upon temperature increases. Notably, it is not clear in wheat whether recombination is also affected upon changes in temperature (Draeger and Moore 2017). Taken together, temperature-induced changes in meiosis alter recombination rates and have detrimental outcomes on both organism fitness and fertility. The potential consequences of these temperature-induced increases in recombination and a potential link between the SC and thermotolerance are further reviewed in Morgan et al. 2017.

Meiosis contains many sexually dimorphic features from the differences in gamete sizes, to the timing of gamete production, to the mechanistic sex-specific changes in establishing the meiotic chromatin and crossing over during prophase I. These sex-specific differences highlight that there is no “one fits all” status for meiosis, and that meiotic phenotypes need to be assayed in both males and females. Future studies analyzing mutants in each sex may reveal important mechanisms in the regulation of key events during meiosis, such as DSB formation, DSB fate, and crossover designation (and licensing).
